# Path integration in a three-dimensional world: the case of desert ants

**DOI:** 10.1007/s00359-020-01401-1

**Published:** 2020-02-04

**Authors:** Bernhard Ronacher

**Affiliations:** Behavioural Physiology Group, Department of Biology, Humboldt-Universität zu Berlin, Philippstr. 13, Haus 18, 10099 Berlin, Germany

**Keywords:** *Cataglyphis*, Desert ants, Path integration, Three-dimensional navigation, Distance estimation

## Abstract

Desert ants use path integration to return from foraging excursions on a shortcut way to their nests. Intriguingly, when walking over hills, the ants incorporate the ground distance, the paths’ projection to the horizontal plane, into their path integrator. This review discusses how *Cataglyphis* may solve this computational feat. To infer ground distance, ants must incorporate the inclination of path segments into the assessment of distance. Hair fields between various joints have been eliminated as likely sensors for slope measurement, without affecting slope detection; nor do postural adaptations or changes in gait provide the relevant information. Changes in the sky’s polarization pattern due to different head inclinations on slopes were ruled out as cues. Thus, the mechanisms by which ants may measure slopes still await clarification. Remarkably, the precision of slope measurement is roughly constant up to a 45° inclination, but breaks down at 60°. An encounter of sloped path segments during a foraging trip induces a general acceptance of slopes, however, slopes are not associated with specific values of the home vector. All current evidence suggests that *Cataglyphis* does not compute a vector in 3-D: path integration seems to operate exclusively in the horizontal plane.

## Introduction

*Cataglyphis* ants are renowned for their path integration capacities. In featureless saltpans individual ants perform large distance foraging excursions (up to 1200 m; Bühlmann et al. 2014; Huber and Knaden 2015), from which they return on a direct, shortcut way to their nests (Wehner and Wehner 1986; Müller and Wehner 1988; Wehner and Srinivasan 2003). Two kinds of information are essential for path integration: information about the direction of any path segment and about the distance covered in that direction. These two variables are then processed to update a ‘home vector’ that represents the homing direction and the distance to the nest (Wehner and Srinivasan 2003; Ronacher 2008). The walking directions are derived primarily via a sky compass that takes advantage of the sun azimuth position and the polarization pattern of the sky (Wehner 1997, 2014; Wehner and Labhart 2006; Wehner and Müller 2006; Heinze and Homberg 2007). Additional cues may be used as well, for example the spectral gradients across the sky (Wehner 1997) or the direction of steady wind (Wolf and Wehner 2000; Müller and Wehner 2007).

Various hypotheses have been proposed how insects may gauge walking distances—energy consumption, optic flow or idiothetic cues (Heran 1956; von Frisch 1965; Ronacher and Wehner 1995; Ronacher 2008). Distance estimates of desert ants are quite precise (Sommer and Wehner 2004; Bühlmann et al. 2014), and surprisingly robust against various disturbances as carrying heavy prey, walking backwards, or even the loss of one or two legs (Steck et al. 2009; Pfeffer et al. 2016). By putting ants on stilts, Wittlinger and colleagues have convincingly shown that *Cataglyphis* uses a pedometer—more precisely a stride integrator—to assess travelled distances (Wittlinger et al. 2006, 2007a; for a similar conclusion in crabs see Walls and Layne 2009). Remarkably, different odometry mechanisms are used by walking and flying insects (Collett et al. 2006). Honeybees use optic flow cues to gauge foraging distances (Esch and Burns 1995; Srinivasan et al. 1997, 2000; Dacke and Srinivasan 2007; Srinivasan 2014) whereas optic flow cues seem less important in *Cataglyphis* ants (Ronacher and Wehner 1995; Ronacher et al. 2000; Bescond and Beugnon 2005), although they can be sufficient to allow distance estimates under certain conditions (Pfeffer and Wittlinger 2016). Although bees cannot use a pedometer during flight, the observation that walking bumble bees do gauge a feeder distance correctly in complete darkness suggests that bees possess a pedometer like ants (Chittka et al. 1999). Honeybees that were induced to walk in a channel system to a 6 m distant feeder indicated path integration in their waggle dances (Bisetzky 1957). This also suggests distance estimation by means of a pedometer. Conceivably, insects may generally employ two distance estimates, optic flow and pedometer based, however with different weights depending on their respective reliabilities (Wolf et al. 2018).

**Fig. 1:**
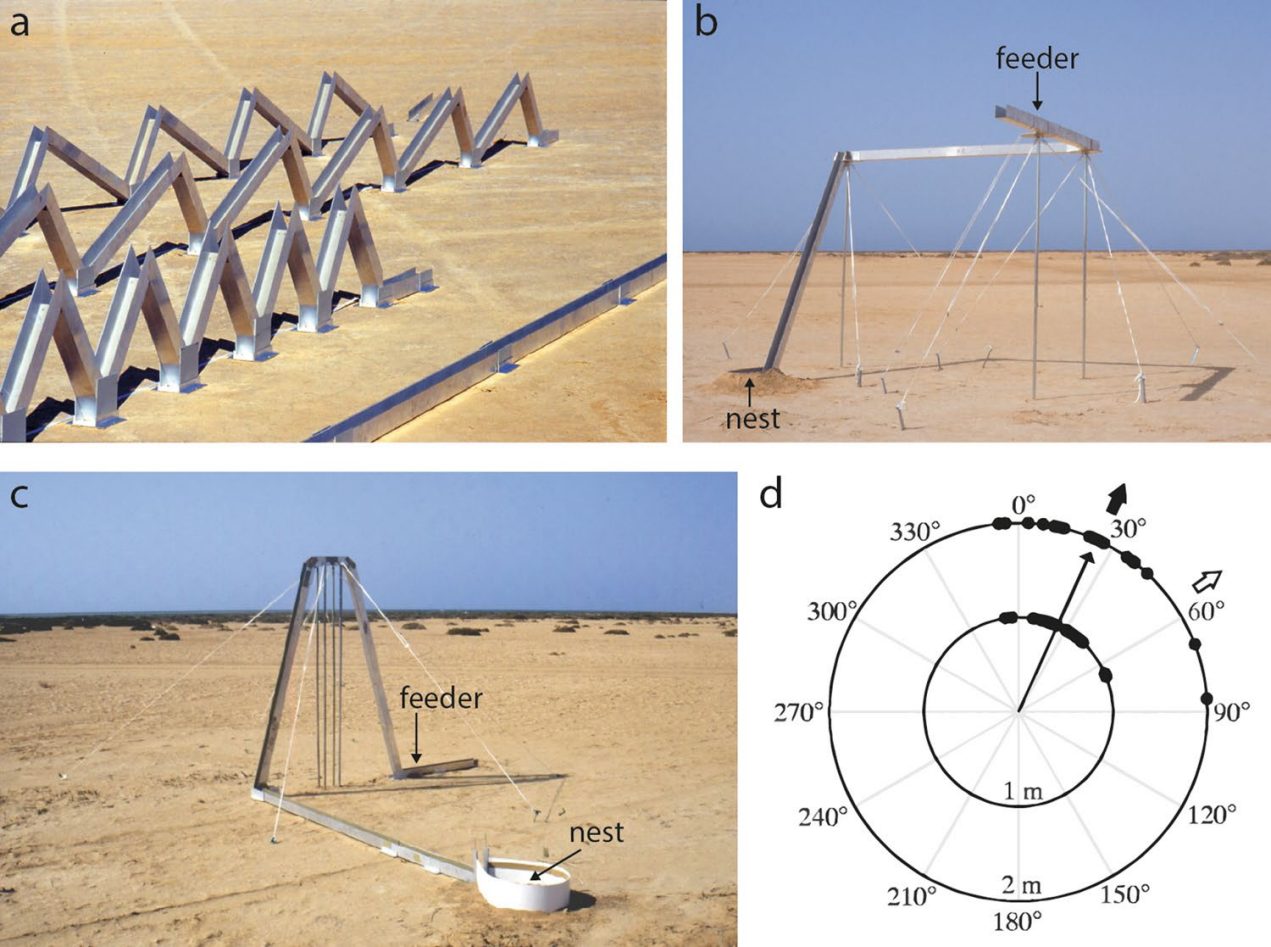
Experimental setups used to test 3-D orientation of desert ants. **a** Linear arrays used by Wohlgemuth et al. (2001, 2002). **b**, **c** Three-dimensional setups used by Maronde et al. (2003) and Grah et al. (2005), respectively. The slopes of the ascent in **b** and of the Λ hill in **c** were 70°. **d** Homing directions of ants trained in setup **c**, captured at the feeder and released in a distant test field. Black arrow: expected direction if ants use the ground distance for path integration, open arrow: expected direction for distance measurement along the whole 3-D path. Long arrow: vector strength at 2 m radius. For more details and additional controls see Grah et al. (2005). **c**,** d** From Grah et al. (2005)

The desert ant, *Cataglyphis fortis*, however, shows another behavioral feat which demonstrates that the measurement of walking distances is even more complex than mere stride integration. In 2000, Sandra Wohlgemuth presented first evidence that ants are able to infer the ground distances when walking over hills (Wohlgemuth et al. 2001, 2002). Having been trained to forage over a series of hills (Fig. 1a), *Cataglyphis* does not only measure the actual walking distance but computes the base line distance, i.e., the projection of the path onto the horizontal plane, and uses this value for path integration. In this respect ants differ from bees which signal to nest mates the total path length when forced to forage on a detour path (von Frisch 1965; Dacke and Srinivasan 2007; Evangelista et al. 2014).

This unexpected capacity of desert ants provoked a series of questions: (1) Do *Cataglyphis* ants compute a three-dimensional vector when travelling in undulating terrain?, (2) What aspects of a three-dimensional path do ants memorize?, and (3) To infer ground distances *Cataglyphis* must be able to measure the inclination of a path and to integrate this information into the assessment of distances. Hence we have to ask what type of cues are used for measuring substrate inclination and how precisely can ants assess slopes?

In this review I will discuss different experimental attempts to answer these questions as well as emphasize some more general aspects of 3-D navigation.

### Do ants perform path integration in the third dimension?

As the experiments described above were performed in a linear array (Wohlgemuth et al. 2001, 2002; Fig. 1a), one could argue, as an attentive referee actually did, that in this design not 3-D but 2-D orientation had been investigated, though in the *x*–*z*-plane. Hence the question of whether *Cataglyphis* does indeed assess ground distances was further investigated in various 3-D path designs (Fig. 1b, c). In the experimental design of Fig. 1c the combination of a 90°-bend of the channel with a hill has the consequence that the path length of the hill as experienced by the ant will be converted into different azimuth angles of the home vector, depending on whether the ants would rely on the ground distance or the actual walking distance (Grah et al. 2005). Clearly, the ants’ mean homing direction corresponded to the ground distance expectation (black arrow in Fig. 1d), and not to the effective walking distance on the hill (open arrow). The data provided an independent confirmation of Wohlgemuth’s results that in hilly terrain the ants indeed rely on the ground distance for path integration—and showed further that ground distance is processed with remarkable accuracy (Grah et al. 2005).

**Fig. 2:**
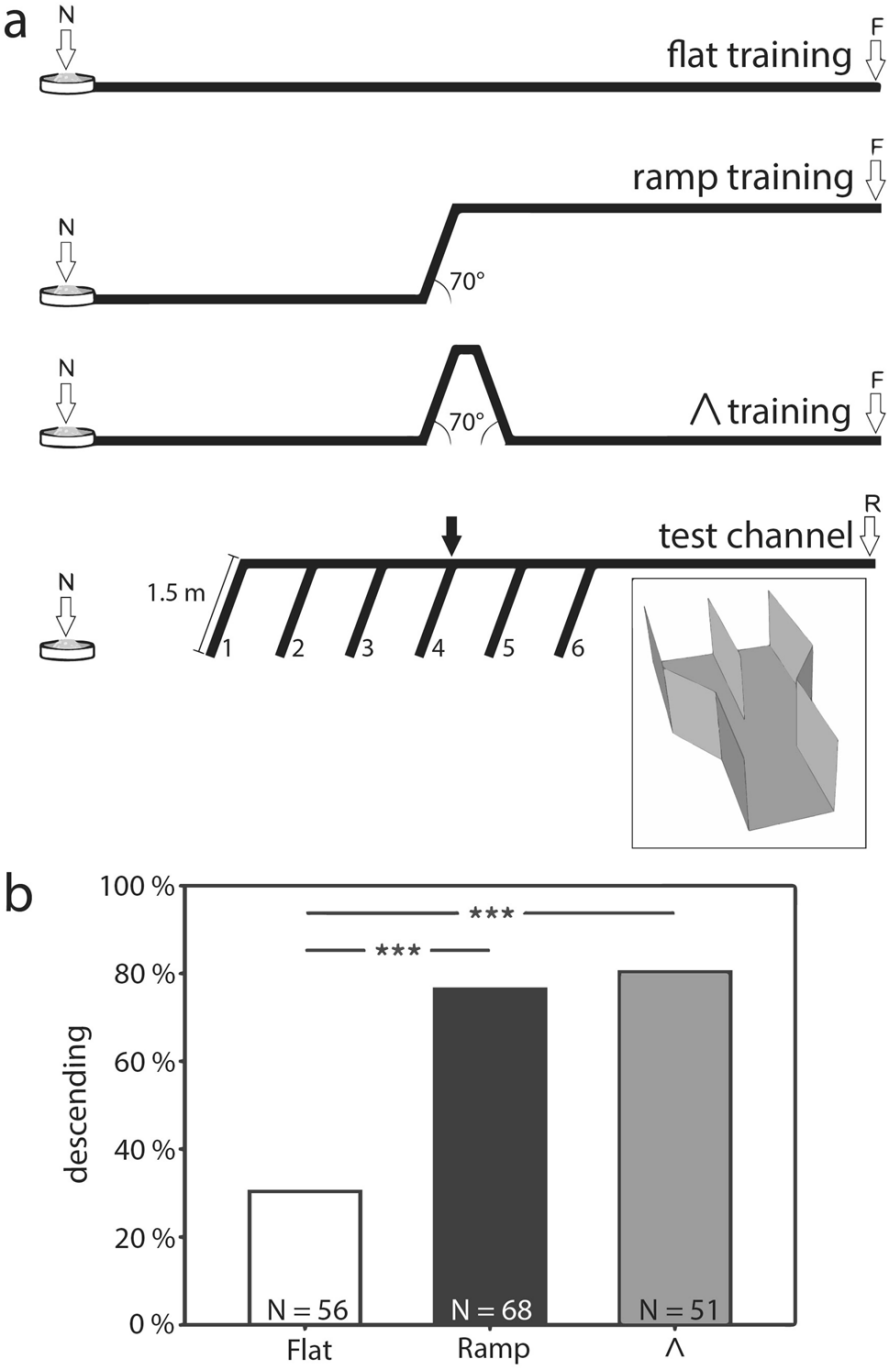
**a** Training and test channels used to investigate 3-D navigation. *N* nest, *F* feeder, *R* release point; arrow indicates the position of the ramp in training (training distances were 12 or 10.5 m, lengths of the ascents 1.95 or 1.5 m). Inset shows the decision points where on one side the channel continued horizontally while the other side led downwards. The downward channels (1.5 m, not shown in the inset) were placed either on the left or on the right side along the whole test channel; the sides were changed daily in a balanced design. **b** Proportion of ants accepting a descent in different training conditions. Ants that showed a U-turn within 20 cm while on the ramp were classified as “rejecters”; after ramp- and Λ-training, the majority of ants showed a complete descent—for the distribution of descent heights and further results, see Grah et al (2007). **a**,** b** From Grah et al (2007)

Due to geometrical constraints the question of whether ants realize a three-dimensional vector navigation is not easy to answer in walking animals, as they allow only limited degrees of freedom in designing training and test setups (Grah et al. 2007). In a first series of experiments one group of ants were trained to visit, via a steep, 70° ramp, a feeder located in an elevated position (Fig. 2a, ramp training). Other ants were trained to forage at a feeder on level ground, one group in a flat channel (flat training), a third group via an included hill (similar as in Fig. 1c) for finally arriving at the level ground feeder (“Λ”*-*training). After several feeder visits the ant’s propensity to accept descents or ascents was tested in test channels offering various potential descents (Fig. 2a, bottom), or ascents (not shown). The expectation was: If ants would compute a 3-D vector, then they should exhibit similar behavior in the “Λ” and flat training—same feeder elevation—but different behavior after ramp training to the elevated feeder. The results of various tests did not conform to this expectation. After “Λ” training the ants accepted test ramps as eagerly as after ramp training whereas ants generally refused to descend or climb ramps after flat training (Fig. 2b and Grah et al. 2007).

1

**Fig. 3:**
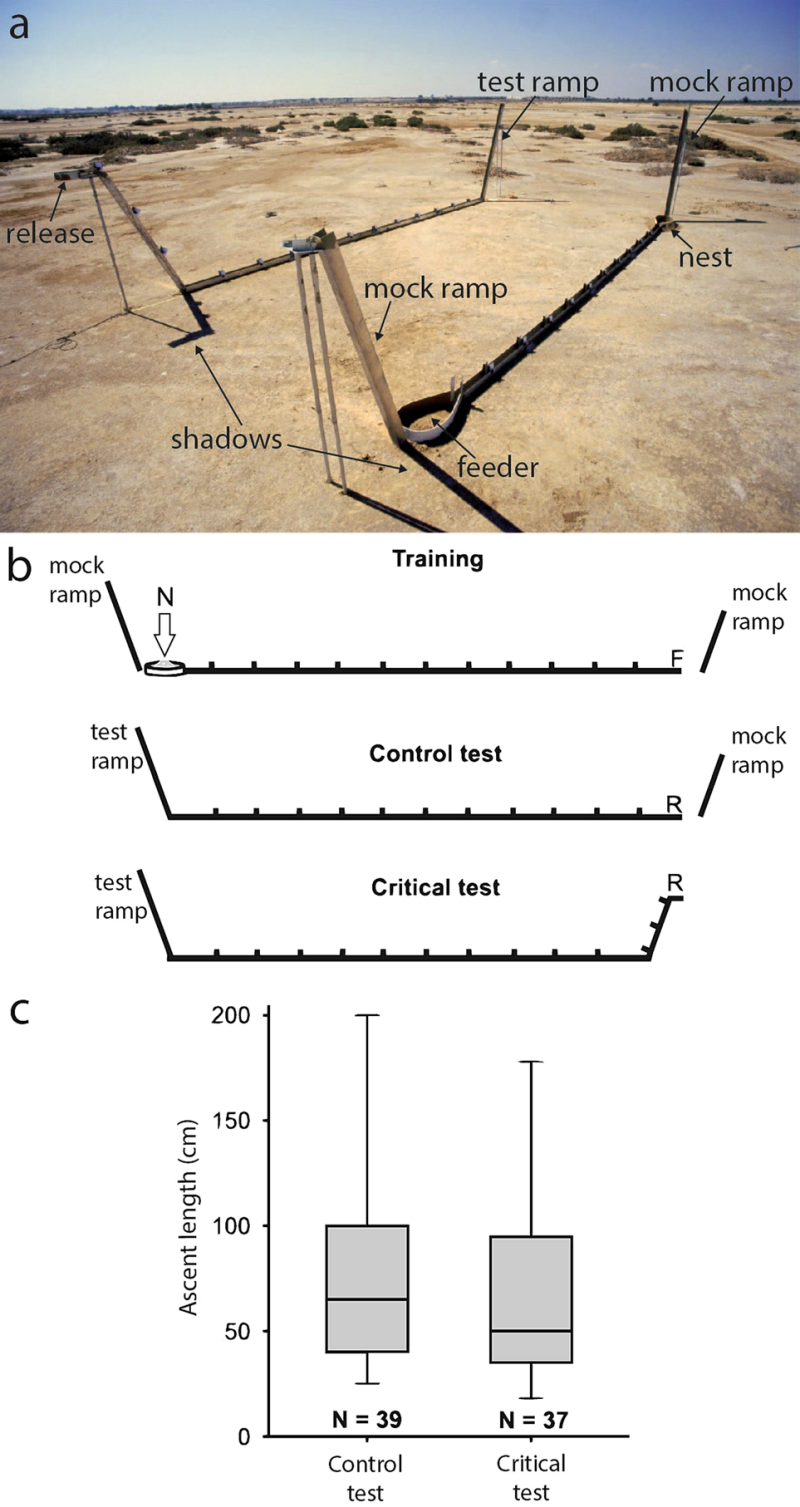
"Half-pipe” experiment as a test for three-dimensional vector navigation. **a** Training and test channels. Ants were trained in a 6 m long flat channel (foreground), provided with small landmarks that were most prominently visible on the homebound way in order that the ants got used to the home path. Near the nest and near the feeder a ‘dummy’ ramp was erected so that later in the test the ants were familiar with these ramps and did not shy away from them. In the test channel the ants could be released either on an elevated platform, from which they had first to descend 1.5 m (critical test), or at the basis of the ramp (control test). At the fictive nest position the test channel ended in a ramp (2 m, 70°) where the ascent height of ants was monitored. **b** Schematic drawing of the experiment, see text; note the different orientation of nest and feeder as compared to the setup shown in **a**; *N* nest, *F* feeder, *R* release point. Note that in the test channel ants could not escape the channel at the test ramp position. **c** Ascent heights of ants released in the control and critical test situations were statistically indistinguishable. **a** Courtesy of Gunnar Grah; **b**, **c** from Grah et al. (2007)

Perhaps the most convincing evidence against the use of a 3-D vector can be deduced from a so-called “half-pipe” experiment (Grah et al. 2007). The idea of this experiment was derived from the observation that—in the horizontal plane—ants reorient their home vector after being forced into a detour (see Fig. 1.1.b in Wehner and Srinivasan 2003). If ants indeed use a 3-D vector, the same vector adjustment should occur if they are forced to a detour in the vertical dimension. Ants were trained in a flat channel to a 6 m distant feeder on level ground (Fig. 3a, b). After at least ten feeder visits an ant was released for its homebound trip at an elevated position in the test channel. Thus the ant had first to descend 1.5 m before reaching the level ground channel; at the fictive nest position the test channel offered a 2 m long, 70° ramp (Fig. 3a). The expectation under the “true” 3-D vector hypothesis was: the descent at the start of the test ramp should induce a negative vertical vector component that—at the end of the test channel—should drive the ant to ascend the ramp unhesitatingly for at least 1.5 m to compensate for this accumulated negative vector component. A different control group of ants was trained in the same way but released on the floor level of the test channel. These ants should concentrate their search for the nest around the basis of the test ramp. Note that in the test channel ants could not escape the channel at the test ramp position. Hence, after flat training an ant searching around the fictive nest position at the basis of the test ramp would likely climb the test ramp for a few decimeters (see Fig. 3c, control). However, contrary to the 3-D vector prediction, both groups of ants exhibited the identical (rather small) ascent heights on the test ramp (Fig. 3c). Note that both groups had experienced a flat training and had never before met a ramp (to exclude the possibility that the test ramp would be treated as an unusual and repellent landmark, mock ramps were erected on both sides of the training channel, see Fig. 3 a, b).

Taking together all different evidence we can conclude that a path integration mechanism functional in all three dimensions is highly unlikely. In desert ants, the path integration mechanism rather seems to operate exclusively in the horizontal plane.

### Which aspects of a 3-D path do ants memorize?

In the experiments comparing ramp training, flat training and “Λ” training, it was tested whether the ants would remember the location of the training ramp, and would select—among a series of offered potential descents—the appropriate one. This was clearly not the case. After ramp training most ants used the first possible descent in the test channel (ramp N° 6 in Fig. 2a) and not the descent at the training position (arrow). Obviously, ants did not link the preference for ramps to certain values of their home vector (Grah et al. 2007).

In a separate experiment, ants experienced a hill segment only on their outbound foraging trip, whereas they were forced to return to the nest in a flat channel. There was no difference in the acceptance of a ramp when ants were tested on their homebound way or—as a control—on their outbound way (Grah and Ronacher 2008). These results further indicate that the ramps were not treated as landmarks. Visual landmarks are stored in the specific context of outbound or homebound trips (Graham et al. 2003; Wehner et al. 2006). Ants also transferred an acquired acceptance of slopes to novel foraging trips in a different direction (Grah 2008; Grah and Ronacher 2008).

To conclude, the encounter of sloped path segments during a foraging trip obviously induces a general acceptance of slopes. However, the slope is not associated with a specific value of the home vector, nor does it have to occur in the sequence as experienced during the outbound foraging trip (Grah et al. 2007; Grah and Ronacher 2008, cf. Kohler and Wehner 2005).

### How could ants assess the inclination of slopes?

The experiments of Wohlgemuth et al. (2001, 2002), and of Grah et al. (2005) have shown that the ant’s path integration module can compensate rather precisely for the increased path length over undulating terrain and that the accuracy of distance gauging is comparable to that on level ground. To achieve this, *Cataglyphis fortis* must somehow measure the slopes of hills and integrate this information into its assessment of walking distances. Hence, the follow up question to ask was: how do ants measure slopes and how precise is this measurement?

#### Energy consumprion as a potential cue?

Lipp et al. (2005) investigated metabolic rates of *Camponotus* ants walking on level ground or on inclines up to 60°. Remarkably, energy consumption did differ only slightly between the five slopes that had been tested (− 60,  − 30, 0, 30, 60°). For small ants the additional costs of vertical transport seem almost negligible. Could energy consumption thus be the parameter of interest to gauge walking distances in uneven terrain? The results reported by Wohlgemuth et al. (2001, 2002) presented further evidence against the hypothesis that ants may use energy consumption to infer traveling distances. Even if ants bore an additional artificial load attached to the pronotum (load on average 1.9 times the ants’ weight) the ants did not misjudge the ground distances when walking over hills (Wohlgemuth et al. 2002; see also Schäfer and Wehner 1993 for loading experiments on level ground). Moreover, the walking speed data also argued against time combined with speed acting as a potential odometric cue (Wohlgemuth et al. 2002).

#### Hairfield proprioreceptors as sensors?

According to Markl (1962, 1964) gravity perception in ants is mediated by hair field proprioreceptors located on the joints between head and thorax, between thorax, petiole and gaster, and on the coxal joints. Hence some of these hair fields might be used to calibrate the odometer to substrate inclination. To investigate the potential contribution of the supposed receptors, Wittlinger et al. (2007b) performed a series of manipulations by shaving hair fields or by immobilizing joints (for example between head and thorax or gaster and alitrunk). Ants were trained in a channel composed of several hills (see Fig. 1a) to visit a feeder located at 6 m ground distance (corresponding to 10 m walking distance), and were tested—in most cases immediately after the manipulation—in a long flat channel. A control group was trained in a 10 m long flat channel and subject to the same manipulations. The expectation was that ants whose graviception system had been impaired would no longer be able to sense the up- and downward slopes and hence search in the flat test channel for the nest at the larger (10 m) walking distance. In stark contrast to this expectation, after various manipulations the ants always focused their nest search at 5 m or smaller distances. Wittlinger et al. (2007b) concluded that the disabled hair plate mechanoreceptors were not the decisive sensors that allow *Cataglyphis* to estimate the steepness of slopes and to derive ground distance.

The only hair plates that had not been accessible to the shaving or immobilizing procedures applied by Wittlinger et al. (2007b), were the sensors on the coxal joints. The potential contribution of this cue was covered in a study by Seidl and Wehner (2008). The authors investigated the kinematics of leg movements of two ant species (*Cataglyphis fortis, Formica pratensis*) which were induced to walk on inclines, and tested several hypotheses of how ants might derive ground distance estimates. A straightforward potential explanation—that the step lengths increased as a consequence of the slope of the substrate—was excluded. There was no gait change and only minor changes in duty factor between swing and stance phases up to inclinations of ± 60°. The authors further concluded that measuring the angular positions of legs (via thorax-coxa joint position sensors) would not help to infer the slope of the path (Seidl and Wehner 2008; see also Wöhrl et al. 2017).

Weihmann and Blickhan (2009) investigated postural adaptations of two ant species walking on inclines. In *Cataglyphis* some compensatory postural changes have been observed, most prominently visible in the caput-substrate angle. However, these changes were not sufficient to guarantee a constant angle of the head relative to the horizontal plane. In contrast, the head positions on slopes changed between approximately horizontal and almost vertical, depending on the inclines and the direction of walking, uphill or downhill (Weihmann and Blickhan 2009).

#### Changes of the polarization pattern?

**Fig. 4:**
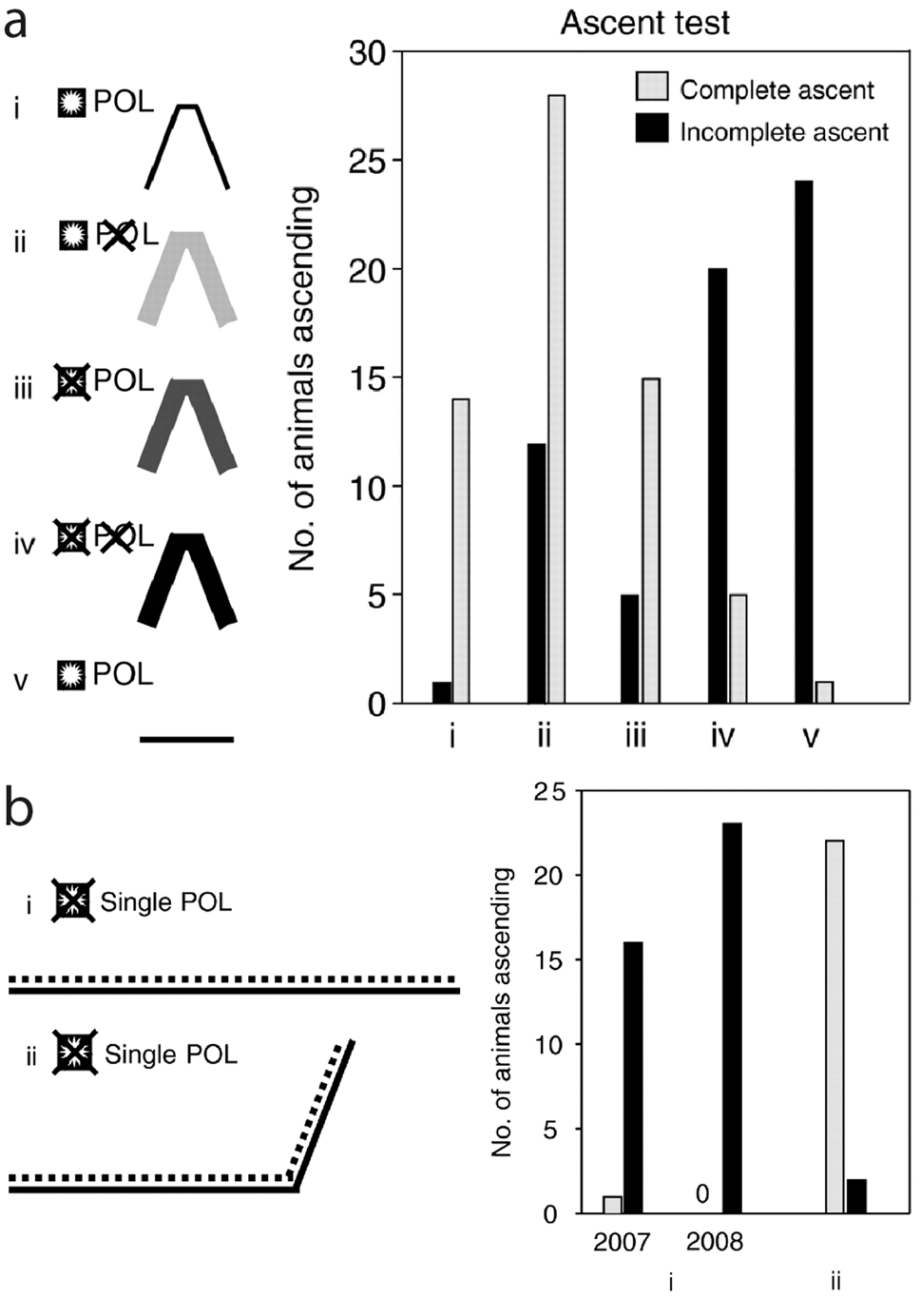
Tests for the potential influence of sky compass information on slope perception. **a** Training in a Λ channel (compare Fig. 2a); the symbols on the left indicate whether sun and/or celestial POL information were available. POL information was excluded by covering the channels with orange perspex filters that blocked UV. In a test channel, now with full sight of the sky, it was monitored whether the ants refused to climb a ramp—i.e., exhibiting a U-turn while on the ramp—or readily accepted the ramp (complete ascent); after training without celestial cues (iv) most ants refused to walk on the ramp, like in the flat control (v); tests in which the propensity to descend was monitored yielded identical results, see Heß et al. (2009). **b** Training and tests in channels covered with a linear POL-filter (left). The dots above the channel indicate the cover with the POL-filter transparency; vision of the sun was excluded. Right: results of the critical experiment: (i) ants trained in a flat channel refused to ascend on the test ramp (black columns) whereas ants of the control group (ii) trained on a ramp readily accepted the test ramp. For further data and controls see Heß et al. (2009), **a**,** b** from Heß et al. (2009)

The observation of markedly changing head positions on slopes (Weihmann and Blickhan 2009) was an incentive to investigate an alternative hypothesis: Whether *Cataglyphis* might monitor changes of the POL-pattern induced by different inclinations of their dorsal rim area (DRA) as a slope indicating cue (Heß et al. 2009). Ants were trained to walk in a channel over a hill with steep slopes (70°; see Fig. 2, Λ training), while either the sight of the sun, or the sight of the celestial POL pattern, or both were excluded (Fig. 4a). Two control groups were trained with full sight of sun and POL pattern, either in a flat or in a hill channel (Fig. 4a, i, v). Remarkably, in contrast to the control groups, ants that had neither sun nor POL-cues while crossing the training hill behaved like ants after flat training—they refused to ascend or descend a test ramp (now with full access to the sky—Fig. 4a, compare iv and v). Previous experiments in the horizontal plane had shown that path segments that did not provide celestial compass cues were ignored by the ant’s path integration system (Sommer and Wehner 2005; Ronacher et al. 2006). This observation was now confirmed for path segments extending in the third dimension (Heß et al. 2009). Similarly, walking bees ignored path segments in which celestial cues were withheld (Bisetzky 1957; see also Chittka et al. 1999). Sky compass cues seem to be a general gate for the transmission of path segments into the integrator.

However, the experiments described above did not directly deal with the hypothesis of interest here. As a next experiment, ants were therefore trained and tested in channels covered with a polarization transparency. The ants thus experienced a single POL direction both in the horizontal and the ascending parts of the channels, which was a sufficient cue for taking notice of these path segments (Fig. 4b). If slope perception—under normal conditions—would depend primarily on a shift of the celestial POL pattern caused by the changed head inclination on slopes, this experiment allows the following prediction: after flat training an ant should now easily accept the test ramp (covered with the POL transparency) since it would not experience any change of the POL pattern that might signal the ramp. The results were quite clear. After flat training—training and tests under the POL transparency—39 out of 40 ants refused to ascend the ramp (Fig. 4b, i); in contrast 22 out of 24 ants readily accepted the slope after ramp training (Fig. 4b, ii). Note that in this paradigm the training in a flat channel was the decisive one. The different responses shown in Fig. 4b, i and ii, demonstrate that a change of the POL pattern cannot be the essential cue for slope perception (for further details and data see Heß et al. 2009).

#### How precise is slope measurement in desert ants?

To explore the discrimination accuracy for different inclinations *Cataglyphis fortis* were trained to visit an elevated feeder via a ramp with fixed slope, and later their spontaneous avoidance of deviating inclinations was monitored. Five training inclinations were used: 0°, 15°, 30°, 45°, and 60°. With this assay ants rejected a ramp that was 25° steeper than the respective training slopes (0°, 15°, 30°); for the 45° training the difference had to be even larger (Wintergerst and Ronacher 2012). It seemed likely that ants are able to discriminate smaller angular differences, but did not show this in the present experimental situation. Sabine Wintergerst then developed a second assay that involved repeated training on two slopes while the ants were mildly punished when choosing the “wrong” slope. The criterion for discrimination was whether a significant proportion of ants did not completely ascend the test ramp (for details of the training and test procedures, see Wintergerst and Ronacher 2012). With this new negative reinforcement procedure the discrimination improved substantially. With training slopes in the range between 0° and 45°, the ants significantly discriminated a steeper test slope that differed by only 12.5°. Intriguingly, after training on a 60° slope, ants readily accepted all steeper slopes, up to a vertical ascent, even after intense avoidance training (Wintergerst and Ronacher 2012). This change of behavior may indicate a saturation range of the sensors involved.

A difference in slope of 12.5° appears rather large but we have to keep in mind that the ant had to remember the training slope and compare the previously stored slope with the actual slope of the test ramp. Of course, one cannot take the behavioral discrimination thresholds of slope inclinations for the minimal sensory jnds (just noticeable differences, sensu E.H. Weber). Nonetheless, probably they indirectly reflect a limit of discriminability.

Using the experimental paradigm shown in Fig. 1c, we can estimate the impact an inaccurate slope measurement would exert on the path integration performance. Under the assumption that an ant would misgauge the inclination by 12.5°, this error would induce only a moderate error angle of the home vector, of less than 7° for slopes up to 45°. If the first leg of the path in Fig. 1c, on even ground, would be longer as compared to the hill segment, the deviation of the home vector would be even smaller. The estimated rather modest impact of the slope measurement error on the home vector suggests that there may have been no strong selective pressure to further improve the accuracy of slope detection. The dramatic behavioral change in slope detection occurring between the 45°- and 60°-training slopes may, however, indicate a physiological limitation, and hence, in future investigations, may give a potential clue to the mechanisms involved in slope perception.

### Comparison with 3-D navigation in other animals

In honeybees the function of the visual odometer was investigated using tunnels of various three-dimensional designs (Dacke and Srinivasan 2007). An analysis of the waggle dances indicated that bees integrated the optic flow cue along the total distance travelled, independent of the orientation of the tunnel (horizontal, vertical, or oblique). Thus, unlike desert ants, honeybees do obviously not extract the horizontal component of image motion in an oblique tunnel. In this respect ants differ from bees which signal to nest mates the total path length when forced to forage on a detour path (von Frisch 1965; Dacke and Srinivasan 2007; Evangelista et al. 2014). Furthermore, the vertical component of a flight path is not encoded in honeybees’ dances (Dacke and Srinivasan 2007). This may be different in stingless bees (*Melipona panamica*) that are reported to be able to communicate the height of a food source (Nieh and Roubik 1998; but see also Hrncir and Barth 2014 for a different interpretation). For jumping spiders it has been claimed that they are able to perform true path integration in three dimensions during the pursuit of prey (Hill 1979).

Although a detailed comparison with results on mammals is beyond the scope of this review, some findings deserve to be mentioned (for reviews see Etienne and Jeffery 2004; Jeffery et al. 2013; Davis et al. 2018). Hayman et al. (2011) compared recordings from place cells and grid cells of rats that walked on a helix-like staircase or climbed a vertical wall with corresponding recordings obtained in flat arenas. The authors found vertically elongated firing fields indicating an anisotropic encoding of three-dimensional space. They suggest “that path integration does not function effectively for movement in a dimension that is perpendicular to the long axis of the animal (such as, for surface dwelling animals, the vertical dimension).” (Hayman et al. 2011, p. 1187). This interpretation, however, has been challenged by Taube and Shinder (2013), see also Ulanovsky (2011). Jeffery et al. (2013) analyze and discuss different ways of how three-dimensional space could be encoded within the central nervous system, and review behavioral evidence as well as neurophysiological studies in three dimensions. The authors favor a coding scheme termed a ‘bicoded map’, with metric properties in the horizontal plane and a non-metric scale in the vertical dimension. Hayman et al. (2015) again question that distance estimation operates in three dimensions. Recordings from grid cells while rats foraged on a tilted surface yielded almost the same patterns as if animals moved on a horizontal plane. The authors conclude that “the neural map of space is ‘multi-planar’ rather than fully volumetric” (Hayman et al. 2015). This view is supported by Porter et al. (2018) who found that place cells in rats’ CA1 region are sensitive to rather small changes in terrain slope, from horizontal to 15° or from 15° to 25° (see also Davis et al. 2018). However, in flying bats the hippocampal representation of three-dimensional space appears to be isotropic (Yartsev and Ulanovsky 2013), corresponding to a ‘volumetric map’ in the terminology of Jeffery et al. (2013). Most recently, evidence has been presented that head direction cells provide a three-dimensional neural compass also in ground-dwelling animals. Thus, a three-dimensional neural map may be a basic general property of mammalian species (Angelaki et al. 2019; Angelaki and Laurens 2020).

## Conclusions and outlook

To pick up the questions posed in the introduction, the experiments reviewed here demonstrated that *Cataglyphis fortis* computes ground distance when walking in undulating terrain, and uses this derived quantity for path integration (Wohlgemuth et al. 2001, 2002). The accuracy with which ants perform path integration even in undulating terrain makes this feat even more admirable (Grah et al. 2005). However, all evidence accumulated so far strongly suggests that *Cataglyphis* does not compute a vector in three-dimensions; rather path integration seems to operate exclusively in the horizontal plane. If an ant encounters a sloped path segment during a foraging trip this triggers a general acceptance of slopes, but the slope is neither associated with a specific value of the home vector, nor does it have to occur in the sequence as experienced during the outbound foraging trip.

The crucial question of how *Cataglyphis* measures the inclinations of the substrate, and how it uses this information to assess ground distances, still awaits further clarification. Several cues proposed so far—proprioceptive sensing of posture, gait, joint angles of the legs and information from the celestial polarization pattern—have been ruled out. A potential “missing link” may be ground reaction force production and complex changes of muscular interactions which depend on substrate inclination (Seidl and Wehner 2008; Wöhrl et al. 2017). Hence muscular force sensors or cuticular strain sensors like campaniform sensilla might provide the crucial cues that allow ants to derive the base-line distance from sloped path segments. Future investigations could focus on a potential contribution of campaniform sensilla or other strain receptors. If it were feasible to disturb the relationship between step length and stepping forces as a first approach, such an experiment may induce misgauged distances and thus help to uncover potential mechanisms involved in slope measurement. Another promising approach could be to extend to inclines the track ball system introduced by Wittlinger and coworkers (Dahmen et al. 2017). This paradigm could allow tracing ant trajectories on much longer ascents or descents while monitoring stepping patterns, potentially in combination with virtual reality environments and neurophysiological recordings.

## References

[CR1] Angelaki DE, Laurens J (2020). The head direction cell network: attractor dynamics, integration within the navigation system, and three-dimensional properties. Current Biol.

[CR2] Angelaki DE, Ng J, Abrego AM, Cham HX, Dickman JD, Laurens J (2019). A gravity-based three-dimensional compass in the mouse brain. BioRxiv.

[CR3] Bescond MT, Beugnon G (2005). Vision-independent odometry in the ant *Cataglyphis cursor*. Naturwissenschaften.

[CR4] Bisetzky AR (1957). Die Tänze der Bienen nach einem Fussweg zum Futterplatz unter besonderer Berücksichtigung von Umwegversuchen. Z Vergl Physiol.

[CR5] Bühlmann C, Graham P, Hansson BS, Knaden M (2014). Desert ants locate food by combining high sensitivity to food odors with extensive cross wind runs. Curr Biol.

[CR6] Chittka L, Williams NM, Rasmussen H, Thomson JD (1999). Navigation without vision: bumblebee orientation in complete darkness. Proc R Soc Lond B.

[CR7] Collett M, Collett TS, Srinivasan MV (2006). Insect navigation: measuring travel distance across ground and through air. Curr Biol.

[CR8] Dacke M, Srinivasan MV (2007). Honeybee navigation: distance estimation in the third dimension. J Exp Biol.

[CR9] Dahmen H, Wahl VL, Pfeffer SE, Mallot HA, Wittlinger M (2017). Naturalistic path integration of *Cataglyphis* desert ants on an air-cushioned lightweight spherical treadmill. J Exp Biol.

[CR10] Davis VA, Holbrook RI, Burt de Perera T (2018). The influence of locomotory style on three-dimensional spatial learning. Anim Behav.

[CR11] Esch HE, Burns JE (1995). Honeybees use optic flow to measure the distance of a food source. Naturwissenschaften.

[CR12] Etienne AS, Jeffery KJ (2004). Path integration in mammals. Hippocampus.

[CR13] Evangelista C, Kraft P, Dacke M, Labhart T, Srinivasan MV (2014). Honey bee navigation: critically examining the role of the polarization compass. Philos Trans R Soc B.

[CR14] Grah G (2008). Die dreidimensionale Orientierung der Wüstenameisen – vereinfachte Repräsentationen von Routen und Räumen: Verhaltensversuche an *Cataglyphis fortis*.

[CR15] Grah G, Ronacher B (2008). Three-dimensional orientation in desert ants: context-independent memorisation and recall of sloped path segments. J Comp Physiol A.

[CR16] Grah G, Wehner R, Ronacher B (2005). Path integration in a three-dimensional maze: ground distance estimation keeps desert ants *Cataghlyphis fortis* on course. J Exp Biol.

[CR17] Grah G, Wehner R, Ronacher B (2007). Desert ants do not acquire and use a three-dimensional global vector. Front Zool.

[CR18] Graham P, Fauria K, Collett TS (2003). The influence of beacon-aiming on the routes of wood ants. J Exp Biol.

[CR19] Hayman R, Verriotis MA, Jovalekic A, Fenton AA, Jeffery KJ (2011). Anisotropic encoding of three-dimensional space by place cells and grid cells. Nature Neurosci.

[CR20] Hayman RMA, Casali G, Wilson JJ, Jeffery KJ (2015). Grid cells on steeply sloping terrain: evidence for planar rather than volumetric encoding. Front Psychol.

[CR21] Heinze S, Homberg U (2007). Map-like representation of celestial e-vector orientations in the brain of an insect. Science.

[CR22] Heran H (1956). Ein Beitrag zur Frage nach der Wahrnehmungsgrundlage der Entfernungsweisung der Bienen (*Apis mellifica* L.). Z Vergl Physiol.

[CR23] Heß D, Koch J, Ronacher B (2009). Desert ants do not rely on sky compass information for the perception of inclined path segments. J Exp Biol.

[CR24] Hill DE (1979). Orientation by jumping spiders of the genus *Phidippus* (Araneae: Salticidae) during the pursuit of prey. Behav Ecol Sociobiol.

[CR25] Hrncir M, Barth FG, Cocroft RB, Gogala M, Hill PSM, Wessel A (2014). Vibratory communication in stingless bees (Meliponini): the challenge of interpreting the signals. Studying vibrational communication, animal signals and communication.

[CR26] Huber R, Knaden M (2015). Egocentric and geocentric navigation during extremely long foraging paths of desert ants. J Comp Physiol A.

[CR27] Jeffery KJ, Jovalekic A, Verriotis M, Hayman R (2013). Navigating in a three-dimensional world. Behav Brain Sci.

[CR28] Kohler M, Wehner R (2005). Idiosyncratic route-based memories in desert ants, *Melophorus bagoti*: how do they interact with path-integration vectors. Neurobiol Learn Mem.

[CR29] Lipp A, Wolf H, Lehmann F-O (2005). Walking on inclines: energetics of locomotion in the ant *Camponotus*. J Exp Biol.

[CR30] Markl H (1962). Borstenfelder an den Gelenken als Schweresinnesorgan bei Ameisen und anderen Hymenopteren. Z Vergl Physiol.

[CR31] Markl H (1964). Geomenotaktische Fehlorientierung bei *Formica polyctena* Förster. Z Vergl Physiol.

[CR32] Maronde K, Wohlgemuth S, Ronacher B, Wehner R (2003). Ground instead of walking distances determine the direction of the home vector in 3-D path integration of desert ants. Proc Neurobiol Conf Göttingen.

[CR33] Müller M, Wehner R (1988). Path integration in desert ants, *Cataglyphis fortis*. Proc Natl Acad Sci USA.

[CR34] Müller M, Wehner R (2007). Wind and sky as compass cues in desert ant navigation. Naturwissenschaften.

[CR35] Nieh JC, Roubik DW (1998). Potential mechanisms for the communication of height and distance by a stingless bee (*Melipona panamica*). Behav Ecol Sociobiol.

[CR36] Pfeffer SE, Wittlinger M (2016). Optic flow odometry operates independently of stride integration in carried ants. Science.

[CR37] Pfeffer SE, Wahl VL, Wittlinger M (2016). How to find home backwards? Locomotion and inter-leg coordination during rearward walking of *Cataglyphis fortis* desert ants. J Exp Biol.

[CR38] Porter BS, Schmidt R, Bilkey DK (2018). Hippocampal place cell encoding of sloping terrain. Hippocampus.

[CR39] Ronacher B (2008). Path integration as the basic orientation mechanism of desert ants. Myrmecol News.

[CR40] Ronacher B, Wehner R (1995). Desert ants *Cataglyphis fortis* use self-induced optic flow to measure distances traveled. J Comp Physiol A.

[CR41] Ronacher B, Gallizzi K, Wohlgemuth S, Wehner R (2000). Lateral optic flow does not influence distance estimation in the desert ant *Cataglyphis fortis*. J Exp Biol.

[CR42] Ronacher B, Westwig E, Wehner R (2006). Integrating two-dimensional paths: do desert ants process distance information in the absence of celestial compass cues?. J Exp Biol.

[CR43] Schäfer M, Wehner R (1993). Loading does not affect measurement of walking distance in desert ants Cataglyphis fortis. Verh Dtsch Zool Ges.

[CR44] Seidl T, Wehner R (2008). Walking on inclines: how do desert ants monitor slope and step length. Front Zool.

[CR45] Sommer S, Wehner R (2004). The ant’s estimation of distance travelled: experiments with desert ants *Cataglyphis fortis*. J Comp Physiol A.

[CR46] Sommer S, Wehner R (2005). Vector navigation in desert ants, *Cataglyphis fortis*: celestial compass cues are essential for the proper use of distance information. Naturwissenschaften.

[CR47] Srinivasan MV (2014). Going with the flow: a brief history of the study of the honeybee’s navigational ‘odometer’. J Comp Physiol A.

[CR48] Srinivasan MV, Zhang SW, Bidwell NJ (1997). Visually mediated odometry in honeybees. J Exp Biol.

[CR49] Srinivasan MV, Zhang S, Altwein M, Tautz J (2000). Honeybee navigation: nature and calibration of the ‘odometer’. Science.

[CR50] Steck K, Wittlinger M, Wolf H (2009). Estimation of homing distances in desert ants, *Cataglyphis fortis*, remains unaffected by disturbance of walking behaviour. J Exp Biol.

[CR51] Taube JS, Shinder M (2013). On the nature of three-dimensional encoding in the cognitive map: commentary on Hayman, Verriotis, Jovalekic, Fenton, and Jeffery. Hippocampus.

[CR52] Ulanovsky N (2011). Neuroscience: how is three-dimensional space encoded in the brain?. Curr Biol.

[CR53] von Frisch K (1965). Tanzsprache und Orientierung der Bienen.

[CR54] Walls ML, Layne JE (2009). Direct evidence for distance measurement via flexible stride integration in the fiddler crab. Curr Biol.

[CR55] Wehner R, Lehrer M (1997). The antʼs celestial compass system: spectral and polarization channels. Orientation and communication in arthropods.

[CR56] Wehner R, Horvath G (2014). Polarization vision: a discovery story. Polarized light and polarization vision in animal sciences.

[CR57] Wehner R, Labhart T, Warrant E, Nilsson DE (2006). Polarization vision. Invertebrate vision.

[CR58] Wehner R, Müller M (2006). The significance of direct sunlight and polarized skylight in the ant’s celestial system of navigation. Proc Natl Acad Sci USA.

[CR59] Wehner R, Srinivasan MV, Jeffery KJ (2003). Path integration in insects. The neurobiology of spatial behavior.

[CR60] Wehner R, Wehner S (1986). Path integration in desert ants. Approaching a long-standing puzzle in insect navigation. Monit Zool Ital.

[CR61] Wehner R, Boyer M, Loertscher F, Sommer F, Menzi U (2006). Ant navigation: one-way routes rather than maps. Curr Biol.

[CR62] Weihmann T, Blickhan R (2009). Comparing inclined locomotion in a ground-living and a climbing ant species: sagittal plane kinematics. J Comp Physiol A.

[CR63] Wintergerst S, Ronacher B (2012). Discrimination of inclined path segments by the desert ant *Cataglyphis fortis*. J Comp Physiol A.

[CR64] Wittlinger M, Wehner R, Wolf H (2006). The ant odometer: stepping on stilts and stumps. Science.

[CR65] Wittlinger M, Wehner R, Wolf H (2007). The desert ant odometer: a stride integrator that accounts for stride length and walking speed. J Exp Biol.

[CR66] Wittlinger M, Wehner R, Wolf H (2007). Hair plate mechanoreceptors associated with body segments are not necessary for three-dimensional path integration in desert ants, *Cataglyphis fortis*. J Exp Biol.

[CR67] Wohlgemuth S, Ronacher B, Wehner R (2001). Ant odometry in the third dimension. Nature.

[CR68] Wohlgemuth S, Ronacher B, Wehner R (2002). Distance estimation in the third dimension in desert ants. J Comp Physiol A.

[CR69] Wöhrl T, Reinhardt L, Blickhan R (2017). Propulsion in hexapod locomotion: how do desert ants traverse slopes?. J Exp Biol.

[CR70] Wolf H, Wehner R (2000). Pinpointing food sources: olfactory and anemotactic orientation in desert ants, *Cataglyphis fortis*. J Exp Biol.

[CR71] Wolf H, Wittlinger M, Pfeffer SE (2018). Two distance memories in desert ants—modes of interaction. PLoS ONE.

[CR72] Yartsev MM, Ulanovsky N (2013). Representation of three-dimensional space in the hippocampus of flying bats. Science.

